# Venturing into host-pathogen interaction in Crimean-Congo hemorrhagic fever to highlight terra incognita

**DOI:** 10.3389/fimmu.2026.1735683

**Published:** 2026-03-06

**Authors:** Samuel M. Shamamba, Jean-Christophe Sabue Mulangu, Amanda C. Horton, Dacquin M. Kasumba

**Affiliations:** 1 Clinical Immunology Division, Institut National de Recherche Biomédicale, Kinshasa, Democratic Republic of Congo; 2 Global Medical Affairs, Ridgeback Biotherapeutics, Miami, FL, United States; 3 Microbiology Service, Department of Medical Biology, Faculty of Medicine, Université de Kinshasa, Kinshasa, Democratic Republic of Congo; 4 Antimicrobial Discovery Development and Diagnostics, Countermeasures Development and Evaluation, UK Health Security Agency, Salisbury, United Kingdom; 5 Axis of Molecular Immunology and Pathogenesis, Molecular Biology Service, Department of Basic Sciences, Faculty of Medicine, Université de Kinshasa, Kinshasa, Democratic Republic of Congo

**Keywords:** antiviral, Crimean-Congo hemorrhagic fever, host, immunity, infection, virus

## Abstract

Crimean-Congo- Hemorrhagic Fever (CCHF) is the most widespread tick-borne disease in the world with a highly variable case fatality rate. It is caused by the CCHF virus (CCHFV). The disease, which has neither approved treatments nor vaccines, has long received very little attention until it was listed as a priority pathogen by WHO. Improving our understanding of mechanisms of host-virus interaction is essential for the development of effective therapeutic and prophylactic strategies. There is still much to be clarified to better understand how the virus interacts with its host and humans. Elucidating these mechanisms will provide insights into viral pathogenesis, immune evasion strategies, and host defense responses. As a result, this will stimulate the development of targeted interventions to mitigate disease severity and improve clinical outcomes. Better understanding of virus characteristics will also improve our surveillance capability which is critical for developing effective pandemic preparedness and outbreak response strategies. Here, we examine the existing landscape concerning the immune response and inflammatory events in CCHFV-human interaction and discuss gaps in our understanding of the disease. Such discussions allow us to highlight priority research directions for the identification of potential targets for improved mitigation approaches or specific therapeutic routes.

## Introduction

Crimean- Congo hemorrhagic fever (CCHF) is most widespread tick-borne disease in world with cases being reported in southeast Europe, Asia, and Africa (1). Due to its potential to cause widespread outbreaks, its severity and lack of specific prophylactic and therapeutic treatments, the disease is considered a priority disease needing urgent attention in terms of research and development (R&D) of diagnostic tools and vaccines (2). Cases can go unnoticed since the disease is usually asymptomatic. However, CCHF can have a wide-ranging fatality rate that can go up to 40%, depending on the affected region and the circulating strain. It was first identified in 1944 among soviet soldiers in Crimea (3, 4) before two other cases from 1956 in the Democratic Republic of Congo (DRC) were linked to the disease (5, 6).

The disease is caused by Crimean-Congo hemorrhagic fever virus (CCHFV), a virus belonging to the *Nairoviridae* family within the class of *Bunyaviricetes*. CCHFV genomic material is composed of single-stranded negative-sense RNA molecules fragmented into three segments (S, M and L) encoding proteins with specific functions. Examples of such proteins include the RNA-dependent RNA polymerase, orchestrating virus replication, and glycoproteins, necessary for virus entry into targeted cells (7). Commonly used broad antiviral drugs such as Ribavirin and Favipiravir target RNA-dependent RNA polymerase activity and have been shown to affect virus replication *in-vitro* and in animal models. In spite of this, these therapeutics lack specificity, causing significant side effects. Sufficient evidence of efficacy against CCHFV in human studies is limited, with conflicting reports. The repurposed drug chloroquine has also been suggested to interfere with virus entry and release in *in-vitro* studies that used human and monkey cell lines (8, 9).

The vectors and reservoirs of CCHFV are arthropod ticks transmitting the virus to vertebrates during blood feed. *Hyalomma* spp. are the main ticks harboring and transmitting the virus. While other tick species are known to host CCHFV, their contribution to natural transmission and circulation of the virus remains unclear. *Hyalomma* spp. are therefore considered the principal virus-transmitting vectors since the endemicity of the disease correlates with geographical regions where this species is common. These ticks are parasites of both domestic (such as livestock) and wild animals (such as ungulates), with immature ticks feeding off smaller animals such as birds and rodents, and fully-developed forms preferring larger hosts such as cattle, horses, goats and humans (10, 11).

Several aspects of the disease, particularly the immunological and pathological profile, are only marginally understood. For example, the mechanism of viral entry and how it escapes host defense measures to establish infection and cause disease still need to be clarified. Similarly, it is not clear which factors result in mild and asymptomatic infection. Moreover, the limited availability of diagnostic tools impedes research and surveillance of the disease in endemic regions. There is, therefore, a strong need for the development of robust serological assays which are readily available for basic serological surveillance which could lead to better characterization of the virus and its disease. Improved molecular characterization of the virus in its different hosts would highlight immunological and virological variations and deepen our understanding of the disease to support therapeutic and vaccine development. Here, we summarize the characteristics CCHF as well as the molecular and immunological features with a particular focus on gaps in the current knowledge. We discuss ways to improve our insight of this infectious disease and to stimulate therapeutic R&D by exploiting immunological events such as antibody-mediated immunotherapy. We do not discuss vaccine candidates as it has recently been discussed extensively (9, 12).

## Structure of CCHFV and genomic mutations

**Figure 1:**
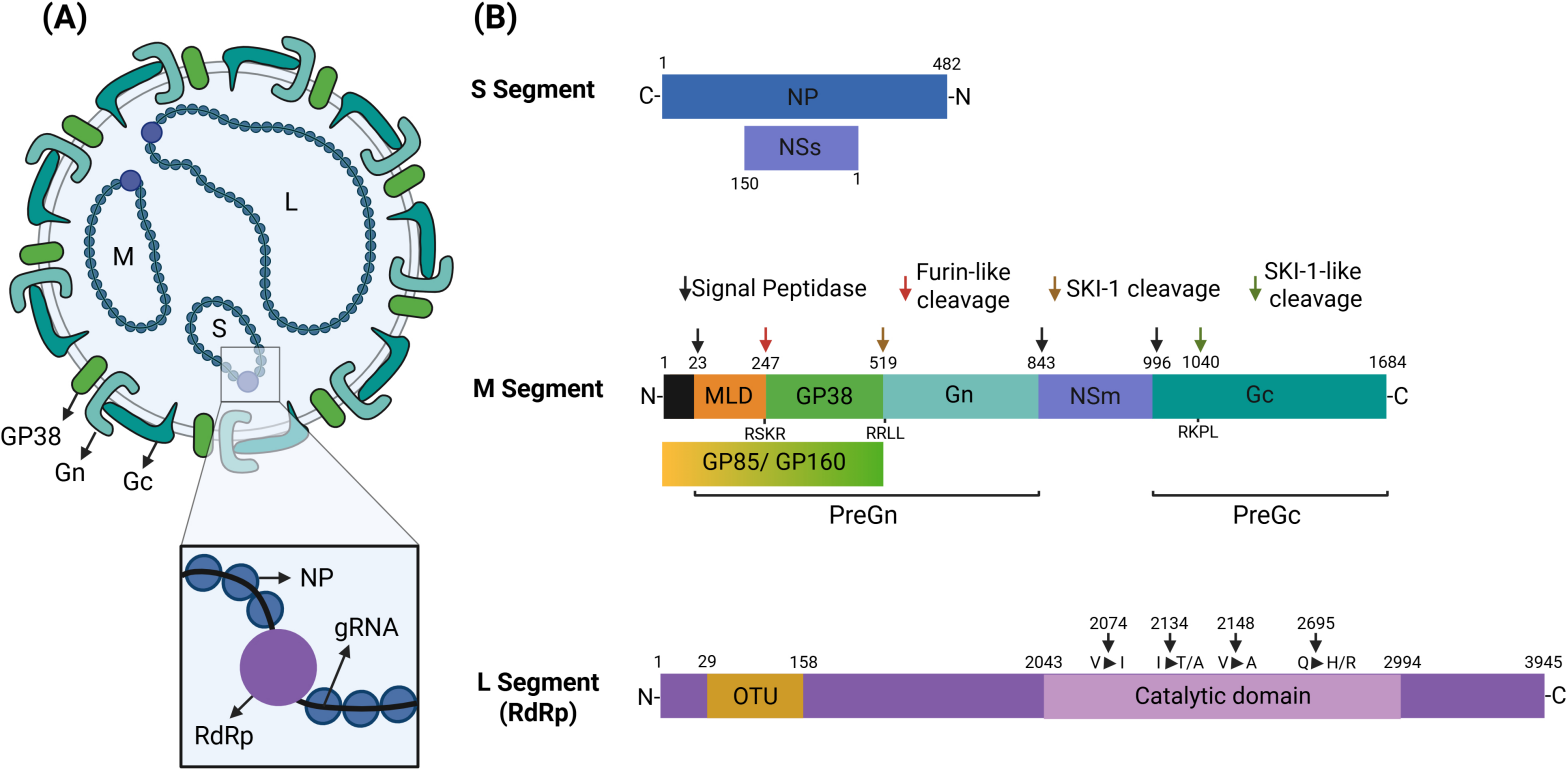
Structure, genomic organisation and proteins of the CCHFV. **(A)** The CCHFV genome is composed of single-stranded negative-sense RNA molecules fragmented as S (small) segment, M (medium) segment and L (large) segment. The nucleocapsid (NP) protein and the RNA-dependent RNA polymerase (RdRp) encapsulates each viral genomic RNA (gRNA) to form three ribonucleoprotein (RNP) complexes. On the viral envelop, glycoproteins Gn, Gc and GP38 form the glycoprotein complex. **(B)** The S segment encodes the nucleocapsid (NP) protein and the non-structural protein NSs. The M segment encodes a polyprotein (glycoprotein) precursor complex (GPC) that is cleaved by host proteases to generate glycoproteins Gn, Gc and GP38, and non-structural proteins GP85, GP160 and NSm. The L segment that encodes for the viral RdRp, which is responsible for RNA replication and cap-snatching, is highly mutated across different genotypes. Four of the frequent mutations (V2074I, V2148A, I2134T/A and Q2695H/R) were found in the RdRp catalytic site (2043–2994 aa) across different genotypes. The N-terminal region of the L protein (29–158 aa) harbours an ovarian tumour (OTU) cysteine protease domain with deubiquitinating and deISGylating activities. The OTU domain is highly conserved, with no evidence of mutations found in the OTU-encoding region of all genotypes sequenced.

All *Bunyaviricetes*, including CCHFV are segmented tripartite negative-strand RNA viruses composed of S, M and L segments, classified according to their nucleotide length (7). While the S segment (approximately 1.7 kb) encodes the nucleocapsid (NP) protein, it also produces the non-structural protein NSs. The NSs protein modulates the host innate immune response by antagonizing antiviral signaling pathways while stimulating cellular apoptosis. These events favor virus replication and contribute to inflammatory events leading to disease symptoms (13, 14). Within the virion, the NP protein encapsulates each viral genomic RNA molecule to form three ribonucleoprotein (RNP) complexes which protect the viral RNA (vRNA) from nucleases (7, 15) (**Figure 1**). This protein is highly conserved and is abundantly produced inside infected cells making it a reliable target for antibody-mediated assays (16). Besides stabilizing RNP complexes, it is unclear whether NP offers other direct advantages to the virus.

The M segment (approximately 5.7 kb) possesses a single open reading frame (ORF) encoding a polyprotein (glycoprotein) precursor complex (GPC) that is cleaved by several host proteases to produce the viral glycoproteins Gn and Gc. While these two glycoproteins are displayed on the viral surface and mediate viral entry, a third one called GP38 is produced in soluble form (17, 18). These glycoproteins are produced as a result of several proteolytic cleavages at various sites of the polyprotein. These proteolytic cleavages allow for the release of glycoproteins in the secretion pathway and the production of infectious virions with matured Gn and Gc on their surface (**Figure 1**). To date, the exact role of GP38 is not fully understood (4, 18). Even so, this glycoprotein is reported to be essential for virus assembly and entry into host cells thanks to its ability to facilitate trafficking of Gn and Gc through the intracellular secretory pathway. This facilitates the integration of Gn and Gc into the virions during the production of infectious particles (19, 20).

Initially thought to be exclusively soluble, recent evidence suggests that GP38 is also found on the plasma membrane of infected cells and on the viral envelope as part of the glycoprotein complex. It is unclear, however, how GP38 (both structural and soluble) contributes to the interaction of the viral glycoprotein complex with host cell receptors to expediate viral entry and infection (17, 21). Furthermore, the production of non-neutralizing GP38-specific antibodies with protective capacity further suggests a critical role of this glycoprotein in virus proliferation which is still to be discovered (17, 19, 22, 23).

As the M-encoded polyprotein precursor GPC is cleaved by different proteases, it produces several other non-structural proteins whose roles are yet to be fully clarified. Cleavage of GPC by signal peptidases (SP), also produce a double-membrane-spanning NSm protein (15kDa) which is suggested to contribute to the trafficking of glycoproteins through the secretory pathway (19). Although its exact mechanism of action is still undetermined, deletion of the NSm domain in the M segment delays viral spread and the onset of disease without a significant effect on viral replication (24). This observation can be directly attributed to impedance in the trafficking of glycoproteins, an essential process for viral egress. Subsequent furin-cleavage of the PreGn also yields a heavily glycosylated mucin-like protein (MLD), another non-structural protein whose role remains unclear. Due to its numerous glycosylation sites, MLD facilitates trafficking of M-encoded polyproteins through the ER-Golgi to ensure delivery of mature Gn and Gc through the secretory pathway (25, 26). However, before cleavage by furin proteases, MLD-GP38 can undergo N-glycosylation in the ER and additional O-glycosylation later in the Golgi to produce GP85 and GP160, respectively. Both GP38 and additionally glycosylated MLD-GP38 forms, GP85 and GP160, are non-structural and are secreted extracellularly as soluble proteins (**Figure 1**) (19, 27). No evidence of GP85 and GP160 being structural proteins exists to date (17, 28).

Ergo, the M segment produces several elements contributing to viral pathogenesis and life cycle (i.e. virus infectivity and egress). Although this offers several antigenic targets for development neutralizing antibodies, the high rate of mutation in the M segment, subsequently making CCHFV the most genetically diverse arthropod-borne virus, constitutes a prohibitively high challenge against the targeting of M-derived proteins for passive and active immunization. This variability is highest in the single open-reading frame (ORF) of the M segment with up to 27% amino acid divergence between strains (19, 29, 30).

Thus far, only Gc, Gn, and GP38-targeting antibodies have been identified and described as antibodies targeting M-derived proteins. Since NSm is intracellular, its capacity to serve as an efficient antigen for neutralizing antibodies is limited. On the other hand, the secreted non-structural proteins containing the MLD domain, GP85 and GP160, would be expected to possess “super antigenic” capacity since they are secreted and circulate within the host. This would make them readily available for antigen-presenting cells and the subsequent establishment of antigen-specific immune responses, as observed with GP38. In fact, non-neutralizing antibodies targeting GP38 and protecting against heterologous challenges in mice have been identified (22, 23, 28). Interestingly, no antibodies targeting MLD alone, which is present in the soluble GP85 and GP160, have been found. Larger immunological studies in populations from diverse geographical regions would provide further insight into the M-derived-mediated immune response including the antibody diversity and help establish its suitability as a therapeutic target.

**Figure 2:**
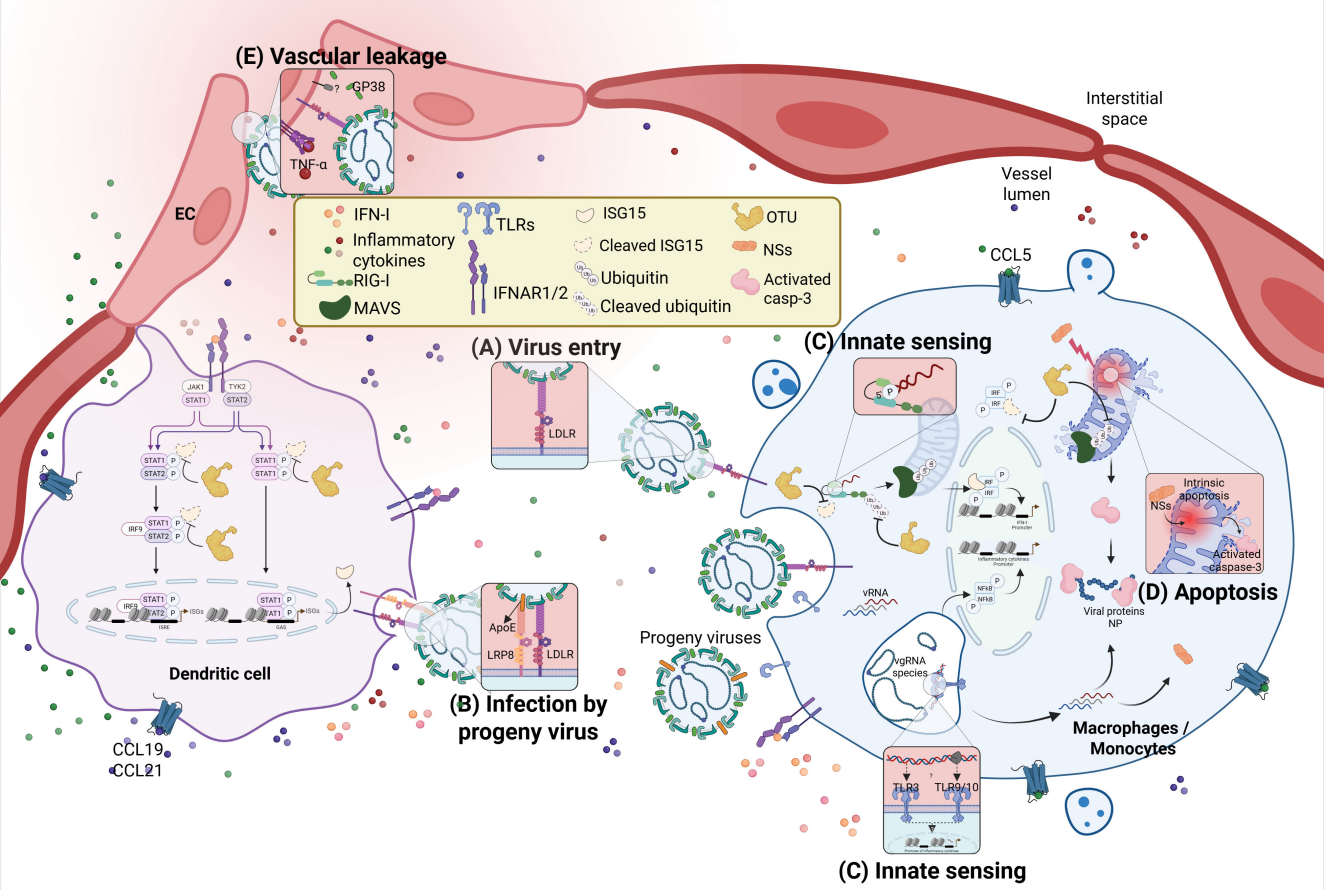
CCHFV interactions with host receptors, innate immune response and inflammation. Mononuclear phagocytes such as macrophages/monocytes and dendritic cells (DC) are the primary cellular target of CCHFV after a tick bite. As infection progresses, the virus spreads to other cell types such as endothelial cells (EC). **(A)** To establish infection, the virus interacts with low-density lipoprotein receptors (LDLR) on the cell surface through its glycoprotein Gc. **(B)** In human hosts, progeny virions incorporate Apolipoprotein E (ApoE) on the membrane of viral particles to enhance infectivity through other host receptors such as Low-density lipoprotein receptor-related protein 8 (LRP8). **(C)** It is unclear how toll-like receptors (TLRs) interact with the virus on the membranes. However, it is speculated that TLRs contribute to inflammatory signatures in symptomatic patients. During cytoplasmic replication, vRNA carrying a monophosphate signature at their 5’ end are sensed by retinoic acid-inducible gene-I (RIG-I) to induce type-I IFN (IFN-) and cytokines through the Mitochondrial antiviral-signalling (MAVS) protein. Signalling of IFN-I through its receptors IFNAR induce JAK/STAT intracellular signalling leading to the production of cytokines and induction of IFN-stimulated genes (ISG), such as ISG15, that help mount an effective antiviral immune response against CCHFV. ISG15 is a ubiquitin (Ub)-like protein that is conjugated to targeted proteins for their activation or inactivation. Together with ubiquitination/de-ubiquitination, ISGylation regulates key signalling molecules in antiviral immunity, including RIG-I and MAVS. The ovarian tumour (OTU) cysteine protease, encoded from the L segment of the virus, cleaves both Ub and ISG15 to reduce RIG-I/MAVS-dependent IFN induction. This will lead to reduced IFN-mediated antiviral response, increased virus replication and disease pathology. **(D)** The viral non-structural protein NSs induce apoptosis of infected cells by disrupting mitochondrial integrity leading to activation of caspase-3. The activated caspases-3 also cleaves viral NP protein. The induced apoptosis contributes to inflammation to amplify pathogenesis in severe CCHF cases. The two proteins NSs and NP, both encoded by the S segment, play key roles in virus interaction with host antiviral factors to down-regulate antiviral immunity and increase inflammation. **(E)** In addition to EC infection, TNF-α produced in response to infection and the soluble GP38 stimulate vascular dysfunction and leakage which in turn favours virus dissemination and inflammation.

The third segment, Large “L” (approximately 12 kb), encodes for the viral RNA-dependent RNA polymerase (RdRp), which is responsible for RNA replication and cap-snatching. Together with the nucleocapsid protein, RdRp encapsidates viral RNA to form functional replication complexes (7). The L protein is highly mutated across different genotypes. Among the known nucleotide changes, four frequent mutations (V2074I, I2134T/A, V2148A, and Q2695H/R) have been observed in the conserved RdRp catalytic site (2043–2994 aa) across different genotypes (**Figure 1**). These mutations are expected to favor viral genetic variability while negatively affecting efficacy of antiviral drugs targeting polymerase activity (31). The N-terminal region of the L protein (29–158 aa) harbors an ovarian tumor (OTU) cysteine protease domain with deubiquitinating and deISGylating activities. This domain plays a pivotal role in immune evasion by suppressing host antiviral signaling (32, 33). Specifically, the OTU domain interferes with the type I interferon response triggered by recognition of vRNA by retinoic acid-inducible gene I (RIG-I) causing attenuation of host innate immunity (34, 35) (**Figures 1**, **2**).

Crimean-Congo hemorrhagic fever virus (CCHFV) demonstrates considerable genetic diversity that closely mirrors its geographic distribution. This diversity is primarily attributed to two mechanisms: the intrinsic high variability within viral populations, and the spread of the virus across different regions and hosts, which facilitates the mixing of strains and thereby increases the likelihood of reassortment and mutation events. The NP, encoded by the S segment, is the most conserved protein across CCHFV genotypes whereas the glycoprotein precursor (GPC) is significantly less conserved, with amino acid identity levels of 75% or lower across strains (9). Genetic diversity in the M segment encoding glycoproteins reflects the genetic adaptation of the virus to selective ecological pressure resulting from adaptation of glycoproteins to various *Hyalomma* spp. found in diverse regions of the world. These arthropods transfer the virus to a wide range of vertebrate hosts. The wide range of hosts in conjugation with the geographical distribution forces the virus to respond to selective pressure in order to establish successful interactions with host cells triggering the genetic adaptation of its glycoproteins (29, 36).

Phylogenetic investigations based on the most conserved segment (S) across strains have established lineages associated with specific geographical distributions. This allowed the classification of genotypes into 5 phylogeographical clades endemic to various regions: clades I to III endemic to Africa, clade IV endemic to Asia, and clade V endemic to Europe. A previously recognized clade VI (Europe) was recently reclassified as a distinct viral species (37). The wide spatial separation of these clusters has been hypothesized to result from the movement of migratory birds carrying infected ticks and the international trade of livestock (38).

As with other RNA viruses, the lack of proofreading activity in CCHFV RdRp contributes to a high mutation rate and increased genetic diversity, with direct implications for virus–host interactions, replication efficiency, and virion assembly. Illustrative examples include amino acid substitutions and point mutations in NP associated with impaired transcription and replication (39–42). Similarly amino acid substitutions in Gc and Gn, MLD of the glycoprotein precursor (GPC), and in the L segment have been described and are implicated in immune evasion and adaptation to diverse hosts (31, 40, 42, 43). Collectively, these molecular alterations underscore the evolutionary plasticity of CCHFV and its capacity to adapt to changing ecological and epidemiological contexts. Our current knowledge of virus biology and events dictating host-virus interaction provide significant insights into its pathogenesis. Nevertheless, additional studies considering broader geographical and ecological niches ought to be undertaken to evaluate variations caused by differences in strains and host characteristics.

## CCHFV interaction with type-I transmembrane proteins

The initial interaction between virus and host cells through its receptors is critical for its capacity to infect the host. For CCHFV, the Low-Density Lipoprotein Receptor (LDLR) has been identified as a key factor for viral entry into humans. This observation has been made using human cell culture systems as well as blood vessels organoids as a more physiologically relevant model of infection (44). A murine model of infection, where type-I IFN response has been blocked to render the mice susceptible to CCHFV, has also been utilized to validate this finding (45). Knocking out LDLR in mice reduced CCHFV in the liver and kidney and delayed the disease in susceptible mice thereby confirming the role of LDLR as an entry receptor in a complete *in-vivo* model (46). LDLR is a type-I transmembrane protein involved in the regulation of cholesterol homeostasis by mediating endocytosis of low-density lipoprotein (LDL) and very low-density lipoprotein (VLDL) (47). During infection, the glycoprotein Gc directly interacts with LDLR thereby triggering endocytosis of the virus to allow its entry into the cell. Evidence suggests that this interaction only occurs with Gc, not Gn (**Figure 2**) (44–46). The contribution of Gn to viral entry is therefore elusive. Uncovering the structure of Gn would allow us to understand the role this glycoprotein plays in the interaction of CCHFV with host receptors.

As the virus replicates inside human cells, it incorporates Apolipoprotein E (ApoE) on the membrane of viral particles to enhance its infectivity through other host receptors such as low-density lipoprotein receptor-related protein 8 (LRP8), another member of the LDLR family (**Figure 2**). ApoE is a ligand of both LDLR and LRP8 (44, 45). Insect cells do not possess ApoE but rather express the functionally similar Apolipophorin-III (ApoLp-III) which also acts as an innate immune receptor (48, 49). As a result, virus progeny produced from tick cells do not use LRP8 (44). Whether or not this host-specific difference might be related to the fact CCHFV uses ticks as a reservoir without causing disease is unclear. The contribution of LRP8 to infectivity is only limited, with LDLR being the main receptor for virus-host interaction through viral Gc (44–46). Evaluating how LDLR mutations influence viremia and disease severity in patients with hypercholesterolemia would provide additional clarification of the critical role this receptor family plays in CCHF pathology within specific populations (50) (51). Although LDLR inhibition and blockade in model mice and in human cells reduces infection, it remains to be seen whether inhibitors of LDLR family can provide therapeutic advantages and improve clinical outcomes in patients (44, 46, 52).

Other membrane-bound receptors, such as Toll-like receptors (TLRs), a family of type-I transmembrane pattern-recognition receptors, have been suggested to play a role in the host response against CCHFV and disease outcome. Although no evidence of direct interaction between the virus and TLRs exists to date, higher frequency of single nucleotide polymorphism (SNP) with variations in the coding sequence of TLRs is associated with disease severity in CCHF patients. For example, the synonymous mutation rs3775290 on TLR3 was found in high frequency in CCHF symptomatic patients (53). There was also a higher frequency of rs4129009 in TLR10 associated with severe cases of the disease (54). All other TLR SNPs described in confirmed CCHF symptomatic patients, including the TLR3 mutation, affected non-coding regions or led to a silent mutation not expected to affect TLR binding and signaling function (53–55). Nevertheless, evaluation of type-I interferon and inflammatory cytokines induction in these patients as a result of TLRs involvement would provide supportive evidence of the role of TLRs in CCHF patients. The ability of variant mutations in patients to influence induction of IFN, IFN-stimulated genes (ISGs) or inflammatory cytokines in response to the infection would constitute evidence of their supportive involvement in the pathology of the disease. It is therefore necessary to clarify mechanisms behind TLRs and CCHF interaction such as the type ligands implicated in the suggested TLR involvements and the contribution of such interaction to immunity and inflammation (**Figure 2**).

Due to the interactions between CCHFV and type-I transmembrane proteins, LDLR is an important contributor to the pathogenesis of the disease. Therapeutic targeting of this interaction in patients is expected to happen in near future (52). Even so, as the virus infects cells through the interaction between its glycoproteins and LDLR, the significance of any interactions between TLRs and the virus is still to be determined while taking into consideration the impact of viral strain and patient geographic diversity.

## RIG-I, the cytosolic sensor of CCHFV, in antiviral response

As the virus enters the host cell and releases its genome, intracellular receptors are engaged to respond to infection. Apart from the confirmed engagement of the LDLR family, the cytosolic pathogen-recognizing receptor (PRR) RIG-I senses intracellular viral RNA species following CCHFV infection. RIG-I binds vRNA species and/or nucleic acid products of viral replication causing a signaling cascade that leads to IFN-I production and response (33, 56). Activation of RIG-I signaling through the mitochondrial adaptor MAVS is therefore critical in hindering the intracellular replication of CCHFV. The antiviral response against CCHFV is mainly dependent on RIG-I rather than MDA5 as the intracellular virus replication produce RNA species that bind specifically to RIG-I. Although the exact identity of the immune response-stimulating RNA species is not clear, the 5’ monophosphorylated viral genomic RNA is speculated to be the RIG-I ligand which induces downstream signaling and cytokine induction (56). MAVS-driven IFN-I production has been shown to be indispensable for antiviral protection in murine models of CCHFV infection, as only mice lacking IFN-I signaling (IFNAR and STAT-1 knockout) were susceptible to infection (**Figure 2**) (57–59). Although these events have only been demonstrated *in vitro* and in animal models, they suggest that TLR signaling contributes very little, if at all, to IFN-I-dependent antiviral response in infected patients. This raises the question of whether the supposed implication of TLR polymorphisms to pathogenesis of the disease could be independent of IFN-I. Perhaps, TLRs would contribute to inflammatory pathways by inducing pro-inflammatory cytokines such as TNF-α in a manner independent of the RIG-I-MAVS axis. This inflammatory response has been tipped to increase to CCHF disease severity, pathogenesis and negative clinical outcomes (57, 60–62). Indeed, CCHFV infection in known to upregulate the receptor-type tyrosine-protein phosphatase R (PTPRR), a phosphatase involved in TLR signaling pathways hence supporting TLR9 and TLR10 involvement in immune response to CCHFV (60).

Our current understanding of the innate immune response to CCHFV highlights the leading role of RIG-I, not TLRs, in sensing infection and inducing IFN-I for antiviral response activation. Whether or not suggested TLR involvements contributes to inflammatory events is still speculative.

## CCHFV antagonizes the antiviral immunity but stimulates inflammation

As part of the IFN-I response, CCHFV infection also upregulates the expression of IFN-stimulated gene 15 (ISG15), a ubiquitin (Ub)-like protein that is conjugated to targeted proteins for their activation or inactivation. Together with ubiquitination/de-ubiquitination, this post-translational modification regulates protein activity. Key signaling molecules in antiviral immunity, including RIG-I and MAVS, require lysine-63 (K63)-linked ubiquitination for their activation or lysine-48 (K48)-linked ubiquitination for their degradation to avoid overactivation of immunity (33). CCHFV encodes an ovarian tumor (OTU) protease which has de-ubiquitination and de-ISGylation activity to modulate host immune response by either dampening or prolonging the activation of signaling molecules (33, 63–66). This OTU, translated from the N-terminal domain of the L-segment protein (**Figure 1**), cleaves both Ub and ISG15, thereby reducing RIG-I/MAVS-dependent IFN induction (**Figure 2**) (34, 35). As a result, this increases virus replication and worsens disease pathology. The capability of the virus to suppress and overcome antiviral immune response provides it with an advantage that could explain why the OTU domain is highly conserved. No evidence of mutations were found in the OTU-encoding region in all genotypes sequenced, while the L segment readily mutated its RdRp catalytic domain (**Figure 1**). Furthermore, CCHFV is the only negative-sense RNA virus which has an OTU with both de-ubiquitinase (DUB) and de-ISGylase activity. The DUB and de-ISGylase ability of the virus could therefore be indispensable for its proliferation (31).

Targeting this critical event in the virus replication cycle provides a novel antiviral target in the search for treatments. This is especially relevant considering that the catalytic domain of OTU is highly conserved across genotypes. However, such pharmacological inhibition of CCHFV’s OTU activity is hindered by the fact that its catalytic domain is highly conserved between humans and viruses (67, 68). Identifying virus-specific “hotspots” for the pharmacological targeting of OTU-dependent DUB and/or de-ISGylase activity is a promising therapeutic option that ought to be explored in greater details.

Furthermore, infected cells undergo apoptosis driven by the viral protein NSs. Apoptosis in CCHFV infection is thought to be a cellular response to inhibit virus replication (14). The non-structural protein NSs disrupts mitochondrial integrity leading to activation of apoptotic caspase-3. The apoptotic activity of NSs depends on its residues Leu-127 and Leu-135 (13). While infection induces the NSs-dependent apoptosis in host cells, the activated caspases-3 cleaves the NP protein, one of the most abundant viral proteins (69). These events are either a host defense mechanism to inhibit CCHFV replication in a manner that is still not fully understood (14, 70) or a decoy mechanism by the virus (9, 69). Conversely, the induced apoptosis could contribute to increased inflammation, which amplifies pathogenesis in severe CCHF cases (**Figure 2**). The two proteins NSs and NP, both encoded by the S segment, therefore play key roles in virus interaction with host antiviral factors to modulate immunity and inflammation.

This indicates that while CCHFV attempts to modulate IFN-I-dependent innate antiviral response to its advantage through its OTU protease, the virus also promotes inflammatory events in the form of apoptosis thanks to its NSs. Due to its well-studied characteristics, pharmacological targeting of OTU proteases in both CCHFV infection and cancer is underway and is considered a promising avenue for treatment. Besides the restoration of an optimal IFN-I response through virus-specific OTU inhibition, the therapeutic potential of blocking NSs-dependent apoptosis through the targeting of residues Leu127 and Leu135 should be explored.

## Type-I IFN and animal models of the disease

Innate immune responses in CCHFV largely rely on type-I IFN as a key mediator (**Figure 2**). However, *in vivo* or clinical investigations evaluating the benefits of type-I IFN as therapeutic options against CCHFV infection is poorly researched. It can be argued that the majority of innate immune characterization has been made using mice models, which do not always reflect human diseases accurately (71–73). For CCHFV infection, the use of IFN-deficient mouse models, specifically IFNAR1-, STAT-1-knockout and mice in which IFN-I has been blocked by the administration of an antibody, are accepted as CCHFV infection models for the study of the disease. These models allow researchers to clarify mechanisms of pathogenesis in severe disease cases and have been used to evaluate therapies and vaccines (18, 23, 37, 57, 59, 74, 75).

Despite this, the use of these immunocompromised mice to evaluate vaccine candidates can be seen to be an inaccurate representation of disease progression within humans as the lack of IFN-I response negatively affects T-cell and B-cell activation and, subsequently, the development of an optimal immune response. When using live virus platforms such as VSV-based vaccine candidates a mouse models deficient in IFN-I response can cause an overestimated vaccine response through the augmented replication of the viral vector in comparison to an immune competent system (76–79). In attempts to overcome this limitation in vaccine studies, transient inhibition of IFN-I signaling through administration of an anti-mouse IFNAR1 into immunocompetent mice has been proposed. This system is expected to block IFN-I response only in the early hours post virus challenge and vaccination, hence allowing the full immune-mediated development of a T-cell response and/or efficient antibodies. Nevertheless, the exact impact of this transient inhibition of IFN-I on early VSV vector replication in this system is still being discussed (80). The particular genetic background of laboratory mice can also introduce artificial differences in response to CCHFV vaccine candidates, as C57BL/6 and BALB/c mice generally display different immune profiles in response to various antigens (81–83).

Limitations associated with using mice models to study CCHF intensifies the need to search for more clinically representative systems for *in vivo* or *ex vivo* studies of the disease to allow for the accurate evaluation of treatments and vaccine candidates. Considering that CCHF is often a mild disease in humans, researchers recently established a cynomolgus macaque model which displayed multi-organs pathology and viremia similar to what is observed during mild to moderate disease symptoms in human. This model offers a favorable alternative to using immunocompromised mice, yet still has limitations as it is unable to provide a complete picture of disease progression in humans that also includes severe cases, particularly ones which result in death (84–86).

In the absence of an animal model which perfectly mimics CCHF pathology and human immune response, currently available models, while still enlightening our understanding of the disease as well as furthering the investigation of mitigation approaches, can cause misleading results. For example, optimal *in vivo* evaluation of IFN-I administration as a treatment is complicated by the lack of an appropriate mouse model since currently available models of infection rely on an IFN-response blockade. For that reason, early attempts to use IFN-I for clinical management of disease within a few patients showed negative results and severe side effects (87). As a result, there have been no further clinical trials. Additional investigations ought to be undertaken as our capacities have advanced since the last clinical trial, using appropriate *in vitro, ex vivo* and *in vivo* pre-clinical models to better characterize optimal conditions (i.e. severe or mild cases, dosage, time and frequency of administration) for IFN-I clinical application.

## Mononuclear phagocytes are the first cellular targets of CCHFV

To date, the underlying mechanisms contributing to the unique susceptibility of humans to CCHFV infection remain unresolved. As a viral hemorrhagic fever, CCHFV is characterized by dysregulated host inflammatory responses. Apart from investigating the role of innate immune markers in CCHF disease, the use of pre-clinical models is indispensable for the study of cellular immunity and inflammation in CCHF. For example, gaining insight into the primary target cells of infection would significantly improve our understanding of cell tropism, virus dissemination and inflammatory events, which in turn support prophylactic and therapeutic research efforts.

In early phases of infection, CCHFV was primarily detected in lymphoid tissues within various organs post infection of murine models. By investigating deeper into the cell populations of IFNAR KO mice lymph nodes (LN) at early stages of infection, it was observed that the virus demonstrates preferred tropisms towards mononuclear phagocytes monocytes/macrophages (**Figure 2**) (58, 88). Whether this tissue and the cellular tropism is driven by specific markers remains to be clarified. Monocytes are driven from the blood to LN for antigen presentation by CCR7-expressing dendritic cells (DC) through CCL5-driven chemoattraction, yet it is unclear whether infected monocytes in peripheral tissues increase CCL5 receptor expression for increased chemoattraction. In the same context, evidence explaining why and how DC would be stimulated to increase production CCL5 for monocytes chemoattraction is still insufficient. It is expected that resident DCs would also be amongst the first cell populations infected by CCHFV following a tick bite, and, as a result of non-self-immune sensing via MAVS and TLRs, they will induce the production of cytokines and increase expression of CCL5 (88–90). To support this, monocyte-derived DC were shown to be permissive to CCHFV infection (91, 92).

As the disease progresses, the virus spreads to other tissues such as the liver and the spleen with a wider cellular distribution affecting phagocytes, antigen-presenting cells and lymphocytes (88). Autopsy investigations detected evidence of antigen-positive or infected mononuclear phagocytes (i.e. monocytes/macrophages and DC) in multiple tissues, including the spleen, liver and intestines (93–95). Mononuclear cells such as B-cells, T-cells and NK cells are not permissive to the virus, hence supporting the suggested preferred tropism of CCHFV towards mononuclear phagocytes (92). In patients with severe disease, examination of the liver has shown a significant number of hepatic lesions associated with antigen-positive Kupffer cells (58, 71, 93). Interestingly, in mice, hepatic macrophages display apoptotic markers regardless of whether they are infected or not (71), suggesting an extrinsic apoptotic pathway, perhaps induced by cytokines, in addition to the described NSs-mediated apoptosis in infected cells (13, 14). Whether or not this extrinsic mechanism driven by inflammatory cytokines is responsible for the well-described depletion of lymphocytes (lymphopenia), a population suggested to be non-permissive to CCHFV, is yet to be elucidated (95, 96). The weakening of the patient’s immune system caused by lymphopenia could also be linked to the failure of the production of specific antibodies observed in severe and fatal human cases (96, 97). Considering that lymphopenia is a hallmark of CCHF and has been associated with negative outcome in patients, further investigation to improve our understanding of this event in haemorrhagic fever diseases is necessary (98).

Once infected, mononuclear phagocytes produce a high concentration of inflammatory cytokines, such as TNF-α, that over-activate endothelial cells (EC) leading to vascular dysfunction which in turns contributes to cellular migration, favouring virus dissemination, and disease pathology (**Figure 2**) (91, 92). Additionally, the soluble glycoprotein GP38 has been suggested to further stimulate EC dysfunction during infection leading to increased vascular leakage in mouse models (99). EC can also be directly infected by CCHFV which induces their activation, as suggested from animal studies and *in vitro* investigations (91, 99). Evidence of EC activation and the resulting vascular dysfunction in disease pathology has been confirmed in autopsies (94, 96). Additional Investigation for means to control EC dysfunction contributing to disease pathology in CCHF would certainly be beneficial for the treatment of severe cases of the disease. For example, the neutralization of key inflammatory cytokines, such as TNF-α, and GP38 could prove to be a synergistic combination to reduce disease severity in symptomatic patients.

Our current knowledge of cellular tropism is based on murine studies and only a few autopsy reports. Analysis of blood leukocytes from patients with either sever or mild disease would provide much needed clarification on the link between virus cellular tropism, disease severity and disease outcome. Furthermore, targeting EC dysfunction and vascular leakage by blocking monocyte/macrophage/DC-derived specific inflammatory mediators could prove to be promising avenue to reduce disease pathology in patients.

## Exploring adaptive immunity in CCHF patients

### CD8+ T cell responses

**Figure 3:**
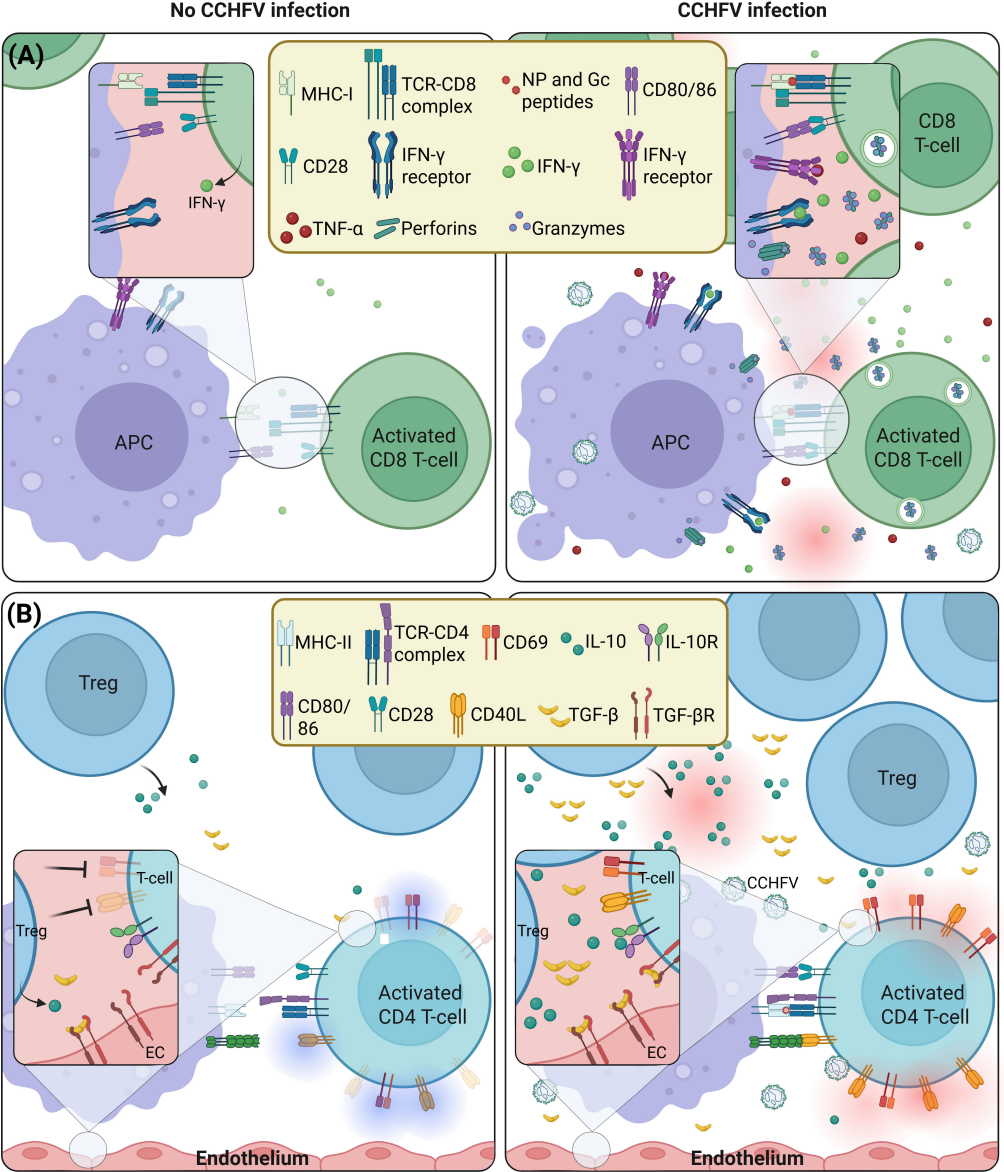
Contribution of T-cells to immune response against CCHFV. **(A)** CD8+ T-cells play an important role in disease pathogenesis, and perhaps disease outcome. CCHFV cases exhibit higher CD8+ T cell number in peripheral blood. As activated CD8+ T-cells induce their effector cytotoxic function, they produce a large amount of IFN-γ and TNF-α in response to CCHFV peptides presentation by antigen-presenting cells (APC). This T-cell effector function is therefore stimulated by specific epitopes in the viral proteins such as NP and GPC that upregulate production of cytokines, perforins and granzymes. **(B)** In CCHFV, the frequency of regulatory T-cells (Treg) is increased while their ability to suppress CD69 and CD154 on activated T-cells is significantly reduced, indicating reduced immunosuppressive activity. Treg frequency and activity contributes to controlling the pathology through the production of anti-inflammatory cytokines, such as IL-10 and TGF-β with minimal effect on cellular activation, represented by CD69 and CD154 suppression.

While CCHFV can antagonize specific innate immune system axes and dysregulating inflammatory pathways (e.g. apoptosis, vascular leakage), our knowledge of the adaptive immunity profile induced by this disease remains limited. Comprehensive understanding of host adaptive immunity in the context of CCHFV infection may help elucidate the mechanisms underlying the variability in clinical outcomes, particularly the distinction between asymptomatic and severe cases. *In vivo* studies using murine models highlighted the critical contribution of CD8^+^ T cells in controlling acute infection. Depletion of this cell population prolonged symptomatic disease and delayed viral clearance (100). However, this observation in mice did not represent human phenotypes previously seen in patients. Fatal CCHFV cases exhibited higher CD8+ T cell numbers in peripheral blood samples but the patient still succumbed to the infection. Increased CD8+ T-cell population alone is, therefore, not sufficient to warrant a favorable disease outcome (101). This CD8+ T-cell effector function is mediated by Gc epitopes that stimulate upregulation of cytokines such as IFN-γ, a key cytokine in antiviral response, and degranulation by this T cells population (102). Interestingly, in humans CD8+ T-cells were also the main producers of IFN-γ in response to CCHFV peptides in an *ex-vivo* study using PBMC isolated from survivors. Although human PBMC responded to both NP and Gc peptides, the latter was a key antigen stimulating this immune effector function on CD8+ T-cells in both systems (**Figure 3**) (102, 103). These observations hint at an important role of CD8+ T-cells in disease pathogenesis, and perhaps also in disease outcome, and highlight the immunogenicity of viral glycoproteins and NP. This stresses the need for further investigations to better understand the contribution of this cell population in the disease as well as in efficacy studies using vaccine candidates.

### CD4+ T cell and Treg responses

Similarly, the contribution of CD4+ T-cells to disease severity and outcome remains elusive. In acute patients, while Treg increased, their ability to suppress CD69 and CD40L (CD154) on activated T-cells was significantly reduced, indicating diminished immunosuppressive activity. While an increased frequency of Treg is expected to prevent the overactivation of the host immune response, the reason for the lower immunosuppressive activity is still unclear (104). The observed increase in Treg frequency in patients correlates with an increase of the anti-inflammatory cytokine IL-10 observed in human cases in a manner that is proportional to disease severity. Treg activity is therefore suggested to contribute to controlling the pathology through anti-inflammatory cytokines with minimal effect on T-cell activation, represented by CD69 and CD154 suppression. Simultaneously, IL-10 helps control Th1 inflammatory response in severe patients (105). While it is hypothesized that Th1/Th2 balance could impact on the pathogenesis and outcome of the disease, there is not yet sufficient clinical evidence to draw any definitive conclusions. Similarly, TGF-β, another cytokine known to be released by Treg to downregulate inflammation, is increased in the blood of severe patients as an attempt to control hemorrhagic symptoms resulting from an excessive inflammation (106, 107). The increased Treg population in patients therefore appears to contribute to immunity against CCHFV through the release of anti-inflammatory cytokines, such as IL-10 and TGF-β (**Figure 3**). Their reduced capacity to suppress overactivation of T-cells has unclear implications on inflammation and/or T-helper activity of T-cells in convalescent patients. These observations warrant additional research to clarify how these events impact on antibody development and long-term immunity against CCHFV.

### B-cell and antibody responses

Human survivors of CCHFV infection efficiently produce IgM and IgG antibodies with neutralization capacity and effector function even years after infection. This suggests a long-term persistence of antigen-specific memory T-cells in CCHF survivors (97, 108, 109). In contrast, in severe cases the patient who succumb to the disease hardly develop antibodies which could explain the need for Treg to suppress over inflammation and correctively stimulate the production of antibodies which would otherwise lead to patient death (96, 97, 110). Antigen-specific immune response, probably through CD8+ T cell effector function, is therefore important in limiting the disease progression during acute infection while Treg helps prevent over inflammation and stimulates antibody production. This antigen-specific immunity persists long after survival thanks to memory T cells and is associated with anti-CCHFV antibodies in survivors which suggests the engagement of B-cells for antibody production (108) (**Figure 3**). In spite of this, evidence profiling B-cell engagement and activation following CCHFV infection to support epitope mapping for vaccine development is still lacking. Investigating B-cell profiles in survivors with neutralizing antibodies would help identify immunodominant epitopes in viral proteins after natural infection, supporting antigenic selection for vaccine development.

Furthermore, how B-cell response and activation is affected in severe and fatal cases to interfere with anti-CCHFV antibody production requires further investigation (96, 97). In the murine model of infection with STAT-1 defected signaling, CD3+ splenocytes T-cell numbers were decreased in infection (59). It is unclear whether this decrease correlates with reported increased apoptotic markers in the blood of patients which in turn correlate with disease severity (111–114). Whether this supposed T-cells apoptosis negatively affects antigen-specific B-cell activation to interfere with antibody production in severe cases remains to be seen in animal models or observed in patients infected with CCHFV.

### Antigenicity and immunogenicity of NP and glycoproteins

**Figure 4:**
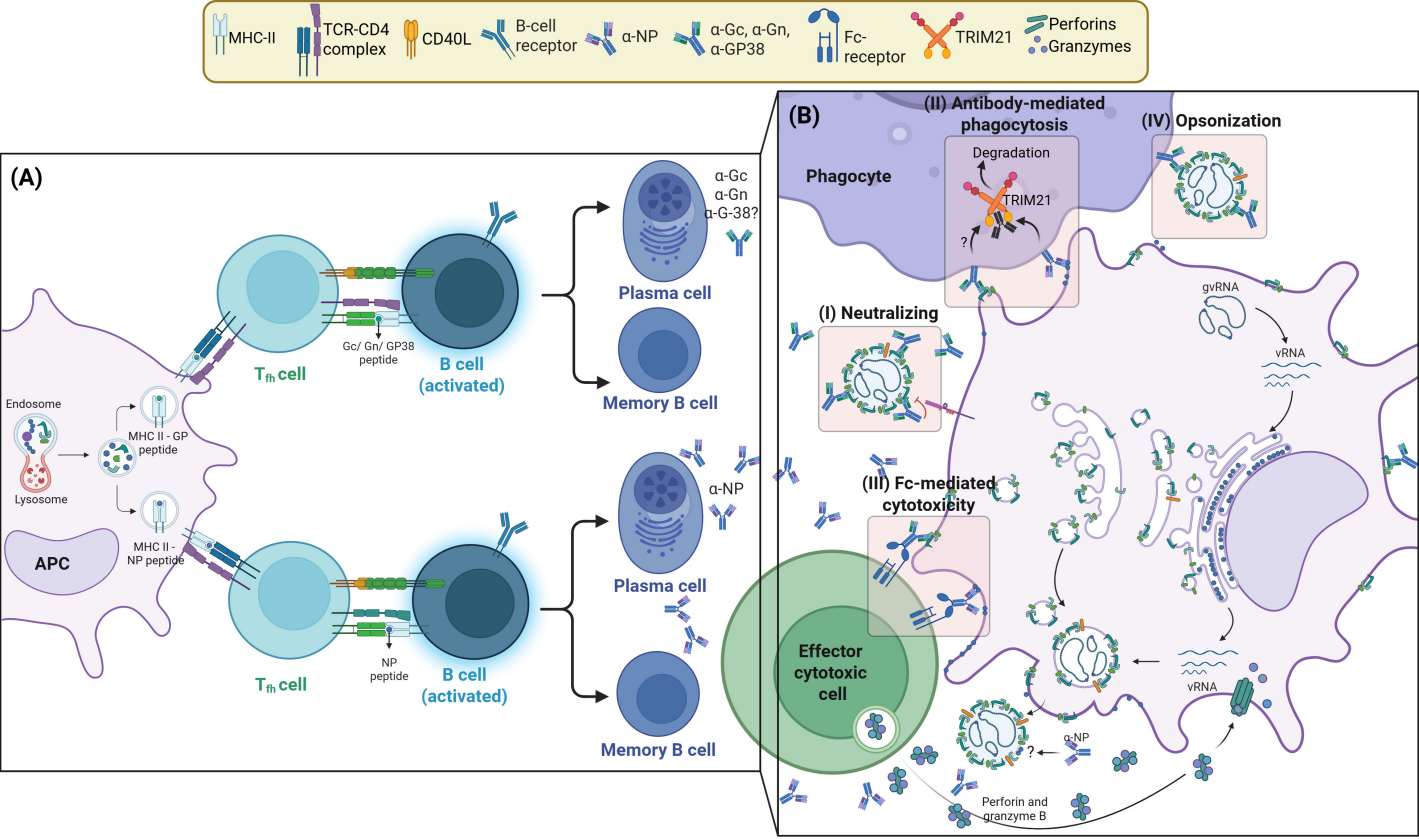
Survivors produce neutralizing and non-neutralizing antibodies providing protection against CCHV infections. **(A)** CCHF survivors develop virus-specific antibodies through B-cells activation. Survivors raise persistent IgM and IgG antibodies against Gc, Gn, GP38, and NP with either neutralization capacity and/or effector function even years after infection. This also suggests long-term persistence of antigen-specific memory T-cells and B-cells in CCHF survivors. The protein NP is among the earliest and most highly expressed antigens upon infection, hence the high level of produced antibodies targeting NP compared to other viral proteins such as α-Gc, α-Gn and α-GP38. **(B)** NP-targeting antibodies have limited neutralizing capacity because NP is predominantly expressed intracellularly in infected cells and is found inside the virion. They cannot prevent viral adsorption or fusion of the viral membrane with endosome, a process driven by glycoproteins (GP). These abundant non-neutralizing antibodies can still provide protection through Fc-mediated mechanisms, such as TRIM-mediated degradation, and Fc-mediated cytotoxicity (that leads to CD8+ T-cells cytotoxicity) to protect against both homologous and heterologous CCHFV strains thanks to NP genetic stability. However, evidence of cell surface-bound NP *in-vivo* is still lagging. Antibodies raised against GPs usually confer protection against homologous challenges. However, α-Gc antibodies isolated from human survivors have demonstrated protection capacity against both homologous and heterologous strains. GP antigens successfully raised neutralizing antibodies. Anti-GPs antibodies are also expected to mount Fc-mediated cytotoxicity, Ab-mediated phagocytosis and opsonization.

Since the NP gene is highly conserved among CCHFV lineages and clades, its application as antigenic target is theoretically promising making it a preferred target in serological assays (16). Moreover, the NP protein is among the earliest and most highly expressed antigens following infection leading to high levels of antibodies being raised against it (**Figure 4**) (16, 108). CCHFV glycoproteins, on the other hand, have more genetic variability between strains making them less attractive targets for immunization against circulating heterologous strains (30), irrespective of the level of Gc-, Gn-, and GP38-specific antibodies in survivors (108). Even so, NP-targeting antibodies are expected to have limited neutralizing and protective capacity. This is because NP is predominantly expressed intracellularly in infected cells and is found inside the virion. These antibodies, therefore, generally lack neutralizing activity since they cannot prevent viral adsorption or fusion of the viral membrane with the endosome, a process driven by glycoproteins. These abundant non-neutralizing antibodies can still provide protection through their Fc-mediated mechanisms (30, 69, 115, 116). For example, in a study on transgenic mice anti-NP antibodies conferred protection through the intracellular FC receptor TRIM21, supposedly after binding to cell surface-bound NP (**Figure 4**). However, evidence of cell surface-bound NP *in-vivo* is limited (69). Additionally, anti-NP antibodies have also demonstrated, in mouse vaccination studies, a protective capacity via cell-mediated immune response. This conferred non-neutralizing cell-mediated immunity was sufficient to protect against both homologous and heterologous challenges, supposedly relying on the genetic stability of NP (80). Human and animal studies showed that CCHFV-specific memory T-cell responses are predominantly directed against the NP protein rather than the glycoproteins (70, 103, 117–119). On the other hand, GPC-expressing vaccine conferred protection only against homologous challenges in murine models, this antigen successfully raised neutralizing antibodies against CCHFV (80).

While neutralizing capacity is an advantage, the ability of NP to induce heterologous protection in animal studies is an important benefit that cannot be overlooked. Vaccines providing such a benefit can protect against various strains of the virus without the need for heterologous boosters or additional strain-specific vaccines. Perhaps a strategy combining both antigens in a single vaccine construct could prove an efficient way to confer virus neutralization capacity, cell-mediated response and heterologous protection (80). Importantly, it remains to be seen if these observations are replicated in NHP models as well as in human trials.

## Investigating virus-specific treatments: monoclonal antibodies for passive immunotherapy

Although CCHFV prophylactic vaccine development has recently benefited from quick advancement, the identification and isolation of monoclonal antibodies for passive immunization is considered an important development toward efficacious treatments post-exposure. This highly specific approach is particularly relevant for hemorrhagic viral diseases with a complicated pathology which affects multiple organs and tissues, such as CCHF. Because of their ability to raise neutralizing antibodies, the search for protective antibodies for passive immunotherapy of CCHFV in humans is preferably focused on glycoproteins. The only evidence of protective antibodies isolated from human survivors of natural infection so far targets glycoprotein antigenic sites in Gc subunit, not NP. These antibodies were neutralizing and conferred protection in mice infected with clades having divergent Gn/Gc sequences and, as a result, constitute the most promising candidate for passive immunization (23). Given the current promising results, deeper analysis, such as epitope mapping, to clarify how this anti-Gc antibody binds to its target to elicit protection against heterologous CCHFV, is warranted. Meanwhile, it can be speculated that this anti-Gc antibody interferes with Gc binding to the viral receptor, LDLR, inhibiting virus entry. Evaluation in non-human primates (NHP) will help elucidate much needed answers and pave the way towards the development of further therapeutic for CCHF. Although this study isolated human mAbs by baiting Gn/Gc binding B-cells, it did not profile antigenic immune preference between NP and glycoproteins for raising of neutralizing and/or protective antibodies in survivors (**Figure 4**). The genetic characterization of B-cells producing protective and/or neutralizing antibodies, irrespective of their targeted antigens, would help narrow down the genomic epitope mapping in naturally developed immunity allowing for rapid identification and development of vaccine candidates. This will also help the characterization of the genomic sequence of protective antibodies allowing for mass production and passive immunization; a strategy previously used with Ebola virus survivors (120, 121). However, the high cost associated with the development and production of therapeutic antibodies remains a challenge, especially for diseases mostly affecting low-middle income countries.

Overall, the glycoprotein Gc displays an attractive immunogenicity as it stimulates neutralizing antibodies in convalescent individuals. These human antibodies have been experimented in mouse models for their passive immunization potential. Although a larger number of NP epitopes activate CD8+ T-cell effector function, including IFN-γ production, in convalescent patients as well as in survivors, Gc peptides similarly stimulate cytotoxic T-cells. For CD4+ T-cells, their immune contribution in CCHF seems to be focused on immune regulation (Treg) through anti-inflammatory cytokine production. It is still unclear which antigens and epitopes predominantly activate CD4+ T-cells in response to infection. Further investigations need to be undertaken to map, with better precision, T-cell as well as B-cell epitopes in exposed individuals in order to narrow the search for vaccines candidates and specific treatments. Additionally, immunological studies targeting different geographical regions with potential genetic diversity both in the virus and in human populations ought to be explored. Doing this will help identify novel immunological targets and additional antibody candidates for the development of specific treatments against the virus.

## Summary and conclusion

The genetic make-up of CCHFV provides advantages to the virus allowing it to produce numerous proteins with specific importance and contribution to its proliferation and capacity to cause disease. While the translated structural proteins from the three segments allow the assembly of progeny virions to maintain infection in the host, multiple non-structural proteins antagonise innate immunity and augment inflammation via various mechanisms, influencing the severity of the disease. These non-structural proteins constitute interesting targets to be explored as treatment options in symptomatic patients. One striking example of such an endeavour is the specific targeting of GP38 by the administration of anti-GP38 antibodies to reduce vascular leakage (99). The challenge remains to fully understand the roles and mechanisms of all these numerous viral proteins, both structural and non-structural in order to design targeted therapeutic approaches.

While the use of antiviral treatments to inhibit the replication of the virus has been put forward in clinical management, their relevance is challenged by the genetic plasticity of CCHFV, relying heavily on its ability to incorporate and fixate biologically significant mutations. The genetic flexibility of this RNA virus allows it to, not only escape targeted antiviral treatments, but also to generate genetically divergent strains as it adapts to different geographical areas and host genetic characteristics. Considering the wide spread of the virus across the globe and the wide variety of potential hosts, with both vertebrates and invertebrates being vulnerable to infection, strengthening our grasp on genetic variations and stability of the virus is vital and calls for real-time adjustments and/or targeting of genetically conserved viral domains. Studies of virus and host characteristics in geographically diverse populations ought to be a research priority.

Our understanding of the interactions between CCHFV and host receptors also needs to be improved to be better exploited for targeted therapeutic strategies. For example, preliminary evaluation of LDLR inhibition in humans to block virus entry is vital. Additionally, our comprehension of how TLRs contribute to inflammation and disease severity is still speculative. Whether or not TLR involvement induces IFN, a crucial contributor to antiviral immunity, is also speculative but considered very unlikely. The application of IFN as therapeutic options in *in vivo* or other pre-clinical models would also clarify whether this key cytokine could prove useful as an antiviral treatment against CCHFV without augmenting inflammation in patients. Even though various *in vivo* models, relying on suppression or blockage of IFN-I pathways, do not perfectly recapitulate human cases, their contribution to the understanding of the disease and evaluation of vaccine and treatment candidates is still incredibly valuable and cannot be dismissed.

Despite this, one important limitation of these immunocompromised mouse models is their reduced ability to fully mount an adequate adaptive immune response. Elements of the adaptive response (specifically cellular immunity and antibody production) are important contributors to disease outcome in CCHF patients. While NP-specific adaptive immunity (cellular and humoral response) can cut across divergent CCHFV strains to provide non-neutralizing protection, GPs-specific antibodies likewise efficiently neutralize the virus. In fact, screenings of antibodies from human survivors identified neutralizing antibodies raised against GP proteins (i.e. Gc subunit) only. Such antibodies provide protection even against divergent strains. This highlights significant insights towards the design of antigens for the ongoing search of efficient vaccines (80). Identified protective anti-Gc antibodies constitute a promising powerful tool for passive immunotherapy, a particularly attractive specific approach to CCHFV therapy (23). Further investigations into host immunity against CCHFV ought to be considered, with a particular focus on identifying additional antibodies capable of protecting exposed populations.

Overall, there is an urgent need for intensified multidisciplinary research and coordinated global efforts to improve disease surveillance, quickly clarify the strain-specific characteristics of CCHFV, and develop effective therapeutics and vaccines, while taking into consideration genetic variabilities. Addressing these challenges is critical to mitigate the growing public health threat posed by CCHFV.
